# Tailored GuideLine Implementation in STrokE Rehabilitation (GLISTER) in Germany. Protocol of a Mixed Methods Study Using the Behavior Change Wheel and the Theoretical Domains Framework

**DOI:** 10.3389/fneur.2022.828521

**Published:** 2022-07-27

**Authors:** Bettina Scheffler, Florian Schimböck, Almut Schöler, Katrin Rösner, Jacob Spallek, Christian Kopkow

**Affiliations:** ^1^Department of Therapy Sciences I, Brandenburg University of Technology Cottbus—Senftenberg, Senftenberg, Germany; ^2^Department of Nursing Sciences and Clinical Nursing, Brandenburg University of Technology Cottbus—Senftenberg, Senftenberg, Germany; ^3^Department of Health Sciences, University of Lübeck, Lübeck, Germany; ^4^Department of Public Health, Brandenburg University of Technology Cottbus—Senftenberg, Senftenberg, Germany

**Keywords:** guideline, implementation, stroke, allied health professionals, rehabilitation

## Abstract

**Objective:**

Evidence-based guidelines are important for informing clinical decision-making and improving patient outcomes. There is inconsistent usage of guidelines among physical therapists involved in stroke rehabilitation, suggesting the existence of a gap between theory and practice. Addressing the German guideline “evidence-based rehabilitation of mobility after stroke (ReMoS),” the aims of this project are (1) to describe the current physical therapy practice within the context of stroke rehabilitation in Germany, (2) to evaluate barriers and facilitators of guideline usage, (3) to develop, and (4) to pilot test a theory-based, tailored implementation intervention for the benefit of guideline recommendations.

**Materials and Methods:**

This study uses a stepwise mixed methods approach for implementing a local guideline. A self-reported online questionnaire will be used to survey the current physical therapy practice in stroke rehabilitation. The same survey and systematic-mixed methods review will be used to evaluate the barriers and facilitators of guideline usage quantitatively. Semi-structured interviews will add a qualitative perspective on factors that influence ReMoS guideline implementation. The Behavior Change Wheel and Theoretical Domains Framework will be used to support the development of a tailored implementation intervention which will be pilot tested in a controlled study. Patient and physical therapy-related outcomes, as well as the appropriateness, such as acceptance and feasibility of the tailored implementation intervention, will be analyzed.

**Conclusion:**

This will be the first endeavor to implement a guideline in German stroke rehabilitation with a focus on changing care provider behavior based on the knowledge of current practice and determining factors using a tailored and theory-based intervention.

## Introduction

In comparison with other diseases, mortality among people after stroke is the second highest of all causes in Europe and the world (1). Stroke typically occurs among older people, but its prevalence among younger adults is also rising (2–4). In the German population, about 2.5% of all adults have already suffered a stroke, and about 200,000 new cases occur every year (5–7). Among neurological disorders, stroke causes 41.2% of the global burden of diseases and is among the most common causes of long-term disability worldwide (8, 9). At about 28 billion euros, Germany contributes 63% of the total disease-related costs of stroke in Europe (10). For the affected individuals, stroke can result in multifaceted dysfunctions (11, 12) and can lead to an impaired physical and mental quality of life over the long term (13). In particular, limitations of motor skills often follow a stroke, and these are crucial for a dependent living with the disease (14, 15). Restoring mobility after stroke is a relevant rehabilitation goal for affected individuals, but only 53% of patients will be able to walk independently outdoors after a stroke (16–20).

Clinical guidelines are defined as “statements that include recommendations intended to optimize patient care. These statements are informed by a systematic review of evidence and an assessment of the benefits and costs of alternative care options” (21). Although guideline-based motor rehabilitation after stroke has been shown to be effective and important for those affected, physiotherapy has been found to deviate from the recommended practice (22–27). Possible reasons for non-adherence to guidelines in acute stroke rehabilitation may be related to healthcare professionals', patients', and healthcare institutions' characteristics, the complexity of some recommended therapies, or complexities in the guidelines themselves (28). In terms of the mobility of patients with stroke, Donnellan et al. noted that guidelines were not specific enough with regard to the performance of the recommended intervention (29). For the purposes of stroke rehabilitation, several guidelines have become available (30). Neurologists and physical therapists of the German Society of Neurorehabilitation (Deutsche Gesellschaft für Neurorehabilitation—DGNR) developed the interdisciplinary “rehabilitation of mobility after stroke (ReMoS)” guideline, which was first published in 2015 by the “Working Group of Scientific Medical Societies” (Arbeitsgemeinschaft Wissenschaftlicher Medizinischer Fachgesellschaften—AWMF) (31). According to the AWMF classification, the ReMoS guideline is considered an “S2e guideline” which implies that the guideline development process is based on a systematic literature search and a systematic selection and appraisal of the evidence. Considering the adverse events and the clinical applicability, the authors recommend therapy goal-directed interventions for improving walking ability, walking speed, walking distance, and the balance of individuals with sub-acute and chronic stroke (31). As the greatest potential for functional recovery in patients with stroke can be expected during the 1st months after disease onset (32), only recommendations for the sub-acute phase during the first 6 months are focused upon here (Supplementary Material 1).

Since the ReMoS guideline was disseminated *via* passive dissemination strategies, and as its publication was not supported by a specific implementation strategy, a gap between theory and practice is to be expected (33, 34). As such, the aims are (1) to describe the current physical therapy practice for stroke rehabilitation in Germany, (2) to evaluate barriers and facilitators of guideline usage, (3) to develop, and (4) to pilot test a theory-based implementation intervention for the benefit of ReMoS guideline recommendations.

## Materials and Methods

### Overview

**Figure 1:**
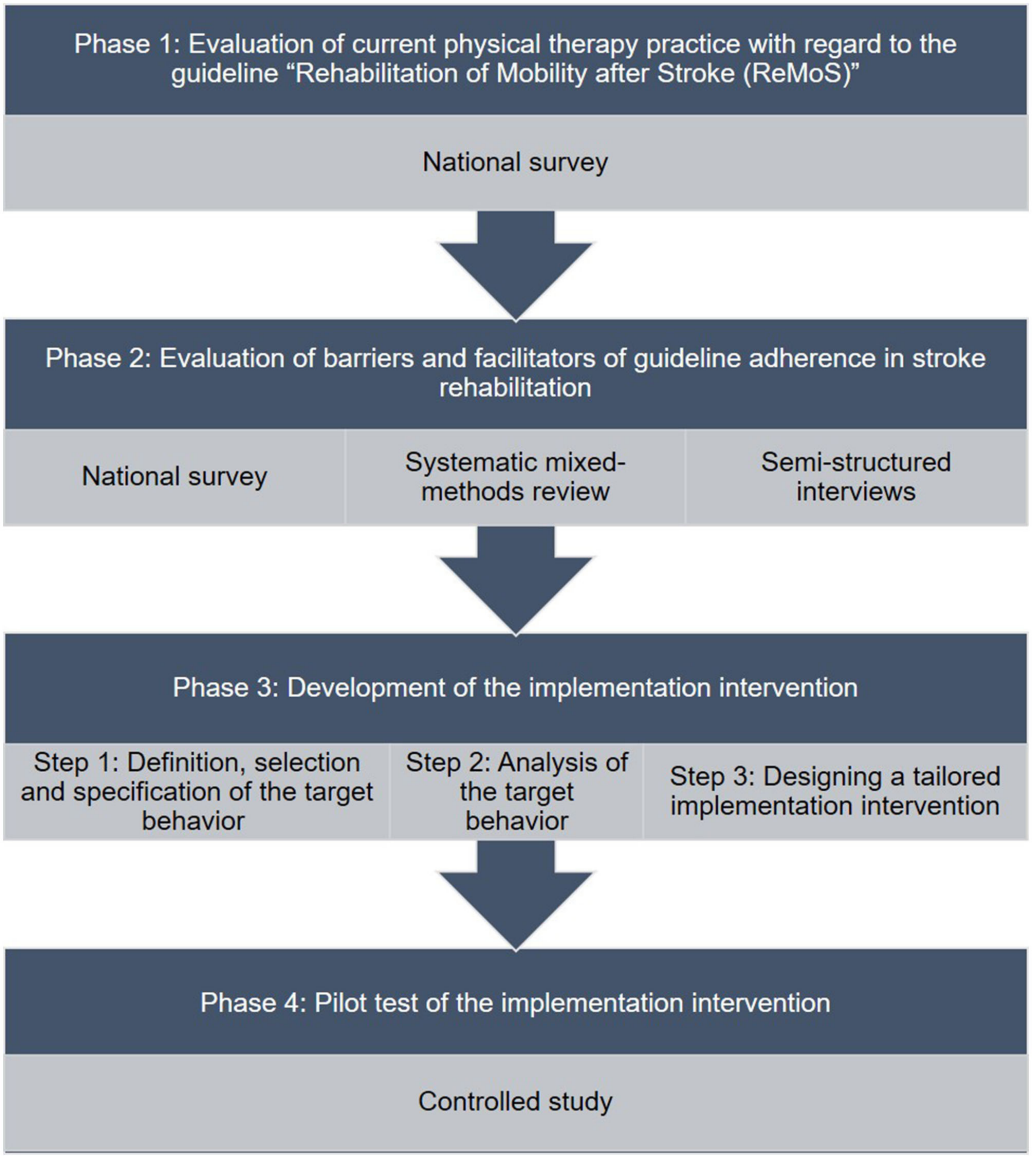
Flow chart of study phases.

This project is divided into four phases guided by the Behavior Change Wheel (BCW) and Theoretical Domains Framework (TDF), which yield a stepwise mixed methods approach to understand and facilitate the use of the ReMoS guideline (Figure 1).

Adoption of clinical practice guidelines by healthcare providers and behavioral changes toward their use is needed to ensure implementation of the guidelines. Theories and frameworks can facilitate the identification of determining factors and can guide the development of tailored implementation interventions. The selection of implementation frameworks should be based on empirical evidence, the applicability within a specific setting, explanatory power, and on the priorities of clinical practice and health policy (35, 36). Although BCW and TDF are comparatively new implementation frameworks, they have already been used to understand and promote adherence to guidelines in healthcare in general (37–39) and specifically within the field of neurorehabilitation (35, 40–42). Both aim to support behavioral changes among healthcare professionals and enable them to become involved in the whole process (43). The BCW was developed from 19 behavioral change frameworks for guiding diagnosis and intervention development with respect to behavioral problems. The core of the BCW forms the “capabilities, opportunities, and motivation—behavior (COM-B)” system (44). To acquire a deeper understanding of a behavior, the TDF, consisting of 14 domains (Knowledge; Skills; Social/Professional Role and Identity; Beliefs about Capabilities; Optimism; Beliefs about Consequences; Reinforcement; Intentions; Goals; Memory, Attention and Decision Processes; Environmental Context and Resources; Social Influences; Emotions; Behavioural regulation), was amended to the COM-B system (45). When a detailed understanding of the targeted behavior is assured, behavioral change techniques will be chosen from a matrix to design the tailored implementation intervention (46, 47).

An implementation shall eventually take place in the “Elbland Rehabilitationsklinik” in Großenhain, Germany (see Figure 1, Phase 3–4). This clinic is a part of the “Regional Working Group Reha Saxony” [Landesarbeitsgemeinschaft Reha Sachsen (LARS) e.V.] and provides capacity for up to 100 multidisciplinary rehabilitation and 25 weaning beds. Following acute care, people after stroke and other neurological diseases receive further care, as regulated by the Federal Working Group for Rehabilitation (Bundesarbeitsgemeinschaft für Rehabilitation, BAR) with regard to the severity of the disease and the rehabilitation goals (48).

### Phase 1: Evaluation of Current Physical Therapy Practice With Regard to the ReMoS Guideline

Since the education of physical therapists in Germany is or has been mostly performed at vocational schools and not at higher education institutions, and since clinical decision-making depends on prescriptions from physicians, knowledge of and adherence to guidelines on the part of the physical therapist is assumed to be limited (49). A nationwide open, anonymous, online survey will be conducted to evaluate the current physical therapy practice in stroke rehabilitation in Germany, as well as to assess adherence to the ReMoS guideline recommendations and other related factors. The reporting of the survey methods and results shall follow the “Strengthening the Reporting of Observational Studies in Epidemiology (STROBE)” statement and the “Checklist for Reporting Results of Internet E-Surveys (CHERRIES)” (50, 51).

The survey will be prepared using the browser-based software “LimeSurvey” and shall consist of three sections with open and closed questions. The first section will include questions for the self-reported use of ReMoS guideline recommendations for the sub-acute phase after stroke, which are relevant to the field of action of physiotherapists. Participants shall rate the frequency of use of the recommended interventions as “never,” “sometimes,” “often,” and “always.” The second section shall relate to barriers and facilitators of guideline use on the basis of the “The Barriers and Facilitators Assessment Instrument” (52). In the third section, sociodemographic data shall be queried. The preliminary version will be drafted according to the recommendations by Dillman et al., and shall be reviewed by the study team and the ReMoS working group (53). Pretesting will be conducted using a convenient sample of physical therapists to check for technical functioning and feasibility using the cognitive technique known as “post-interview probing” (54). The survey will be open to (1) trained physical therapists, who (2) currently work in sub-acute stroke rehabilitation in Germany, and who have (3) sufficient reading and writing abilities in the German language. Information on the purpose and extent of the survey as well as the means of data storage shall be provided in advance. Participation will be voluntary, and no incentives shall be offered. If participants leave their e-mail address with the intention to participate in further study sections, these shall be stored separately from the survey data. To access the survey, every participant must approve written informed consent and self-reported eligibility criteria. An adaptive answering system shall be used to limit the burden on participants. Snowball sampling will be used to disseminate the access link to the online-survey *via* e-mail, a QR code, and social media. The data will be analyzed descriptively using R and R Studio version 1.0.143 (or newer versions of “The R Project for Statistical Computing,” Vienna, Austria) for all questionnaires that are answered at least in part (confirmation of the eligibility criteria and one further item). Each survey item will be analyzed using descriptive statistics as appropriate. Guideline adherence will be defined as 80% of the participants complying with the ReMoS guideline recommendations either “always” or “often” (55).

### Phase 2: Evaluation of Barriers and Facilitators of Guideline Adherence in Stroke Rehabilitation

#### Systematic Mixed Methods Review

International insights to barriers and facilitators of guideline use in stroke rehabilitation will be acquired by conducting a systematic review according to the guideline for mixed methods systematic reviews from the Joanna Briggs Institute (JBI) (56). The trial registration number on PROSPERO is CRD42020160258. The “Preferred Reporting Items for Systematic Reviews and Meta-Analyses (PRISMA)” statement will guide the reporting of the review (57).

The aims of this review are (1) to synthesize barriers and facilitators of guideline adherent physical therapy and (2) to identify at which level physical therapy is performed according to clinical practice guidelines within the context of stroke rehabilitation. The Cochrane Database of Systematic Reviews, the Cochrane Central Register of Controlled Trials, MEDLINE/PubMed, the Physiotherapy Evidence Database (PEDro), and the Database of Research Into Stroke (DORIS) will all be screened for published studies. Additionally, study protocols will be searched in clinicalTrials.gov, clinicaltrialsregister.eu, and the German Clinical Trials Register. Studies published or registered in the English or German language are considered eligible for inclusion. Studies that will be included are (1) reports on physical therapy during inpatient or outpatient stroke rehabilitation, which (2) present barriers or facilitators of the guideline adherent to physical therapy (3) and/or refer to the level of physiotherapy guideline adherence as an outcome, or which feature either a qualitative or quantitative study design or a mixed methods design that allows a clear extraction of quantitative and qualitative components. Studies investigating the abovementioned outcomes during the acute phase after stroke will not be included. Eligible articles will be independently screened by two reviewers. If any disagreements occur, a third reviewer will be consulted. Full texts will be managed using the JBI “System for the Unified Management, Assessment and Review of Information (SUMARI)” (58). An adapted version of the “JBI Mixed Methods Data Extraction Form following a Convergent Integrated Approach” will be used. If there are any missing information, the corresponding author of the primary study will be contacted. Included studies will be appraised using the “Mixed Methods Appraisal Tool” (59). To analyze the primary outcome regarding barriers and facilitators, a convergent-integrated approach will be used. The above approach enables the synthesis and integration of quantitative and qualitative study results by converting quantitative data into a qualitative format (60).

#### Semi-structured Interviews

Semi-structured interviews are used to provide a qualitative approach to acquiring additional understanding of the factors influencing the ReMoS guideline under the conditions of the German healthcare system. The reporting will follow the “Consolidated criteria for reporting qualitative research (COREQ)” paradigm (61).

A convenient sample of at least 10 physical therapists in Germany will be recruited by e-mail invitations given out to inpatient and outpatient rehabilitation facilities, *via* social media, on the website of a professional association, and *via* professional contacts. To gather a broad range of information, a diverse sample will be recruited with respect to gender, age, professional education level, duration of working experience, professional role, and healthcare setting (inpatient or outpatient rehabilitation). An interview guide will be drafted, revised by the study team, and pilot tested (Supplementary Material 2). The interviews will focus on factors influencing guideline usage in daily physical therapy practice and shall be conducted in the German language. Probing questions related to the TDF domains will be used to acquire further information. Interviews will be conducted either in a one-on-one setting, personally, or *via* phone/video call and shall take no longer than 45 min to complete. Participants shall receive and approve a written informed consent before taking part in the interviews and the interview guide shall also be provided in advance. The first author will conduct the interviews, unless it transpires that she has a personal relationship with the interviewees. All interviews shall be audio recorded and transcribed verbatim. Sociodemographic data shall be collected afterward. Data collection and storage shall be done pseudonymously. Transcripts from the interviews will be returned to the participants if desired for revision within 2 weeks. For data management and analysis, MAXQDA Analytics Pro (release 20.0.08 or later) shall be used. Inductive thematic analysis will be used to develop themes (62). Two team members shall carry out the initial coding of data from the first interview independently. The coding scheme shall be consented to and the first author shall code all the other interviews iteratively. Finally, the themes will be mapped against the TDF and shall be identified as barriers or facilitators with respect to ReMoS guideline usage.

### Phase 3: Development of the Implementation Intervention

#### Step 1: Definition, Selection, and Specification of the Target Behavior

Results from the earlier study components (survey, systematic-mixed methods review, and semi-structured interviews) shall lead to a list of candidate target behaviors, which might potentially influence the usage of the ReMoS guideline. Members of the “Elbland Rehabilitationsklinik” in Großenhain, Germany, the study team, and the ReMoS working group shall rank the candidate target behaviors independently as to whether they appear to be promising in terms of their impact on ReMoS guideline usage, their likelihood of being changed, their spillover effect on related behaviors, and whether they seem to be easily measurable. Out of the top-ranked candidate factors, one or two shall be chosen as target behavior(s). The target behavior(s) shall then be specified further (who, when, where, how often, and with whom, see Figure 1: Phase 3, Step 1).

#### Step 2: Analysis of the Target Behavior

Focus groups shall be queried to study target behavior(s) regarding their occurrence and to identify what needs to be changed within a specific setting (see Figure 1: Phase 3, Step 2). The reporting of this study component shall follow the “Consolidated criteria for reporting qualitative research (COREQ)” document (61).

Two to three focus groups with 6–10 participants taken from a relevant sample of, e.g., management, administration, and health professionals of the “Elbland Rehabilitationsklinik,” shall be implemented (63). Participants who shall be sought are (1) ≥18 years old and (2) directly or indirectly involved in the target behavior(s). Mixed groups shall be invited to promote diversity and represent a wide range of opinions/information levels and to keep the burden for the clinicians as low as possible. The moderated focus groups shall be queried during the working hours in the clinic environment on the basis of a semi-structured discussion guide. The guide shall be developed with open-ended questions to determine what needs to be changed regarding the target behavior. The focus groups shall be audio recorded and a member of the study team shall make field notes. All participants shall receive and approve a written informed consent before taking part in the group discussion and shall be asked to provide sociodemographic information. The focus group discussions shall be transcribed and managed using the software MAXQDA Analytics Pro (release 20.0.08 or later). Thematic analyses shall spawn themes according to the TDF, and the results shall allow a “behavior diagnosis” to assist in the development of a tailored implementation intervention to improve the use of the ReMoS guideline (62).

#### Step 3: The Design of a Tailored Implementation Intervention

The development process for the tailored implementation intervention shall be undertaken according to the BCW guidance and in close collaboration between stakeholders of “Elbland Rehabilitationsklinik” and the study team (see Figure 1: Phase 3, Step 3).

Regarding the Affordability, Practicability, (cost-) Effectiveness, Acceptability, Side-effects/Safety, and Equity (APEASE) criteria, appropriate intervention functions (e.g., education, environmental restructuring, enablement, feedback, persuasion, or training) to address findings from focus groups shall be selected in discussions (44). Since an intervention design at the policy level is beyond the scope of this project, policy categories shall not be considered to be relevant as recommended by the BCW guidance. Most appropriate behavior change techniques (e.g., feedback regarding behavior, verbal persuasion, or social support) that match the selected intervention functions shall be selected from the Behavior Change Theory Taxonomy, v1 (BCTTV1) after appraisal consistent with the APEASE criteria (44). Finally, the mode of delivery (e.g., *via* face-to-face or remote mode, individual or group mode) shall be chosen. The reporting of the final implementation intervention shall follow a structured scheme (64).

### Phase 4: Pilot Test of the Tailored Implementation Intervention

A controlled study design will aim to evaluate the effectiveness, appropriateness, acceptability, and feasibility of both the tailored implementation intervention and the implementation process in order to improve ReMoS guideline usage (65). The trial was registered on the German Clinical Trials Register with the number DRKS00019024. The reporting of this study will follow the “Standards for Reporting Implementation Studies (StaRI)” statement (66).

Routine practice shall be monitored in a single-center design in the “Elbland Rehabilitationsklinik” over a control period of 6 months. During this time, healthcare professionals shall not receive any instructions to change their habits or follow the ReMoS guideline recommendations any differently than they had before. Afterward, the implementation phase including the tailored implementation intervention shall be carried out (6 weeks). This will then be followed by a further monitoring period of 6 months. The implementation intervention shall be provided to all therapists involved in motor rehabilitation, regardless of whether they participate in the study.

Healthcare professionals shall be eligible for participation if they are (1) ≥18 years old, (2) trained therapists providing motor rehabilitation, and (3) continuously employed during the study period. Patients will be eligible to participate if they are (1) ≥18 years old, (2) diagnosed primarily with stroke, and (3) having improvement in mobility as one of their activity-related rehabilitation goals. Reasons for exclusion include other diseases of organ systems that negatively impact on motor rehabilitation (e.g., other severe neurological or musculoskeletal conditions, acute renal or cardiac diseases and other pre-existing disorders that potentially reduce the rehabilitation potential). A study nurse shall screen patients for eligibility. Written informed consent shall be provided and approved before the start of the study for each participant. Primary implementation outcomes will be the German versions of the Acceptability of Intervention Measure (AIM), the Intervention Appropriateness Measure (IAM), the Feasibility of Intervention Measure (FIM), and ReMoS guideline adherence (healthcare professional level) (67). Secondarily recommended core-outcomes will be used for further evaluation at the patient level within 1 week after the start of rehabilitation, at the week of discharge, and 6 months after discharge for the health-related quality of life among people with stroke (PROMIS® Scale v1.2 - Global Health) (68–70). Recruitment and retention rate as well as compliance with the intervention protocol shall be evaluated at the study feasibility level (71). As the present study represents a pilot study for testing the feasibility, acceptability, applicability, and possible effectiveness of a targeted implementation intervention, the number of cases required to determine significant effects has not been determined beforehand. Data analysis as well as the blinding of patients, healthcare providers, and assessors will not be possible because of the controlled before-after experimental design and the active intervention measures. In order to reduce the risk of systematic bias, staff and patients will only be informed in detail about the content and objectives of the implementation study if this is explicitly requested (72). A between-group analysis will be used to evaluate pre-post changes to indicate whether the intervention is successful with regard to the primary healthcare professional and patient outcome.

## Discussion

Studies have been conducted both internationally and in Germany on the implementation of the guidelines for and changes to stroke rehabilitation, although with uncertain effects (73–76). Although the ReMoS guideline was first published in 2015, knowledge of the guideline is lacking in Germany, as is its usage. Since the release of the guideline was not accompanied by a tailored dissemination or implementation strategy, this project aims to pilot test a tailored implementation intervention for the purposes of promoting this guideline.

As we attempt to implement the ReMoS guideline, our first task is to explore current physical therapy practice and to describe a hypothetical rift between guideline recommendations and current clinical practice. Based on a comprehensive study of determining factors, where multiple methods have been used, a tailored theory-based implementation intervention is to be designed in close collaboration with stakeholders. This will be done using the BCW guide (44) and will be tested in a controlled study for feasibility and possible effects on clinical practice and patient outcomes.

As a restricted implementation of recommended interventions can contribute toward avoidable disabilities and indeed harm (77), guideline implementation is relevant for any health condition (78, 79) and for any healthcare profession (80, 81). Barriers to guideline-compliant practice have been found to be individual, external, or guideline related, and these have been studied both in general (82–84) and in specific circumstances (85, 86). Knowledge of existing barriers, as well as the use of theoretical underpinning of behavioral change, is recommended to develop promising strategies for addressing the factors that determine guideline adherence (87). Even so, different frameworks are used in healthcare implementation research (88, 89), meaning heterogeneous results have been observed regarding effectiveness (90–92). With regard to physical therapy practice specifically, multifaceted and tailored interventions seem to be the most promising types (43, 93, 94).

The BCW and TDF have been successfully applied to develop and tailor implementation interventions by healthcare professionals in the context of stroke rehabilitation (41, 42, 95, 96). Two recent studies from Australia have given grounds for optimism regarding the design of tailored interventions, guided by the BCW and TDF in the field of guideline implementation of stroke rehabilitation. Both studies reported the feasibility and acceptance of the intervention and Jolliffe et al. cited a possible effect on guideline adherence in the hands of occupational and physical therapists (75, 97).

### Limitations

Neurorehabilitation services in Germany differ from others both in Europe and the world, which will probably impact on current practice, perceived barriers, and facilitators, the applicability of the implementation intervention, and the transferability of our results. The reasons underlying this might lie with the specifics of the respective rehabilitation systems and the comparatively low proportion of academically trained physiotherapists in Germany (98).

Despite the effort we expended on selecting the theoretical underpinnings, issues might still arise. Both the BCW and the TDF are validated and commonly used in implementation research, but to date, only the BCTTV1 has been translated into German (47). Here, a systematic procedure is used, but subjective and pragmatic decisions are still required. Further issues might also arise due to the design of the study components, since results rely in part on self-reported data and on limited blinding modalities and are subject to possible contamination.

## Conclusion

The project outlined here will be the first to a use a behavioral change approach to promote guideline adherent practice among healthcare professionals working in stroke rehabilitation in Germany. We will gain an understanding of ReMoS guideline usage, the factors that determine usage, and the feasibility of a tailored implementation intervention to change clinical practice. In addition, this project shall also inform recruitment, retention, and the power of future studies to evaluate the actual effect of the intervention using a randomized study design. Understanding of current practice and theory will guide this project toward implementing a local guideline in an inpatient stroke rehabilitation setting. This study shall provide information on the feasibility of a tailored implementation intervention, possible outcomes for clinical practice and patients, and the realization of future effectiveness studies.

## Author Contributions

All authors have contributed to the study concept and design. BS and CK conceived the study, substantially contributed to the development of the study design, have given relevant intellectual input, and drafted the manuscript. AS, KR, FS, and JS gave input relating to the development of the study design. All authors read, improved, and approved the final manuscript.

## Conflict of Interest

The authors declare that the research was conducted in the absence of any commercial or financial relationships that could be construed as a potential conflict of interest.

## Publisher's Note

All claims expressed in this article are solely those of the authors and do not necessarily represent those of their affiliated organizations, or those of the publisher, the editors and the reviewers. Any product that may be evaluated in this article, or claim that may be made by its manufacturer, is not guaranteed or endorsed by the publisher.

## Abbreviations

AIM: Acceptability of Intervention Measure
APEASE: Affordability, Practicability, (cost-) Effectiveness, Acceptability, Side-effects/Safety and Equity
AWMF: Arbeitsgemeinschaft Wissenschaftlicher Medizinischer Fachgesellschaften
BAR: Bundesarbeitsgemeinschaft für Rehabilitation
BCW: Behavior Change Wheel
BCTTV1: Behavior Change Theory Taxonomy (v1)
CHERRIES: Checklist for Reporting Results of Internet E-Surveys
COM-B, Capabilities: Opportunities and Motivation-Behavior
COREQ: Consolidated criteria for reporting qualitative research
DGNR: Deutsche Gesellschaft für Neurorehabilitation
DORIS: Database of Research Into Stroke
FIM: Feasibility of Intervention Measure
IAM: Intervention Appropriateness Measure
JBI: Joanna Briggs Institute
LARS: Landesarbeitsgemeinschaft Reha Sachsen
PEDro: Physiotherapy Evidence Database
PRISMA: Preferred Reporting Items for Systematic Reviews and Meta-Analyses
PROMIS: Patient-Reported Outcomes Measurement Information System
ReMoS: Rehabilitation of Mobility after Stroke
StaRI: Standards for Reporting Implementation Studies
STROBE: Strengthening the Reporting of Observational Studies in Epidemiology
SUMARI: System for the Unified Management, Assessment and Review of Information
TDF: Theoretical Domains Framework.
