# Supplementary material for: Tailored GuideLine Implementation in STrokE Rehabilitation (GLISTER) in Germany. Protocol of a Mixed Methods Study Using the Behavior Change Wheel and the Theoretical Domains Framework
Source: Front Neurol. 2022 Jul 27;13:828521. doi: 10.3389/fneur.2022.828521 (PMC9363877; doi:10.3389/fneur.2022.828521)
Supplement: Supplementary file 1 [file Table_1.docx]

Supplementary Material

Supplementary Table: English translation of positive therapy-goal directed recommendations of the “Rehabilitation of the Mobility after stroke (ReMoS)” guideline on the sub-acute phase after stroke. Adapted from (Dohle et al., 2015).

| **Level of recommendation** | **ReMoS guideline recommendations for the sub-acute phase after stroke** |
| --- | --- |
| **Achieving ability to walk in non-ambulant patients** | |
| **A** | - |
| **B** | Intensive walking training, where possible with end-effector device |
| **Achieving ability to walk in (partially) ambulant patients** | |
| **A** | - |
| **B** | Intensive walking training: conventionally *or* using a treadmill (preferred progressively) |
| **Improving walking speed in (limited) ambulant patients** | |
| **A** | Task-oriented endurance training (using progressive treadmill training) *or* progressive circuit training |
| **B** | - Intensive walking training: without treadmill *or* - Intensive walking training using a treadmill *or* - Intensive supervised home exercise (strengthening, endurance, balance) with progression - Walking training with stimulation of the flexor reflex afferents |
| **Improving walking distance in (limited) ambulant patients** | |
| **A** | Task-oriented endurance training |
| **B** | - Intensive supervised home exercise (strengthening, endurance, balance) with progression - Intensive walking training using a treadmill, especially progressive, aerobic treadmill training |
| **Improving balance (static, dynamic, falls)** | |
| **A** | - |
| **B** | - Intensive walking training without treadmill *or* - Intensive walking training using a treadmill *or* - Intensive supervised home exercise (strengthening, endurance, balance) with progression - Motor-relearning-Programme |

Legend: A: shall be applied; B: should be applied
