# Supplementary material for: Tailored GuideLine Implementation in STrokE Rehabilitation (GLISTER) in Germany. Protocol of a Mixed Methods Study Using the Behavior Change Wheel and the Theoretical Domains Framework
Source: Front Neurol. 2022 Jul 27;13:828521. doi: 10.3389/fneur.2022.828521 (PMC9363877; doi:10.3389/fneur.2022.828521)
Supplement: Supplementary file 2 [file Data_Sheet_1.docx]

Supplementary Material

**Interview guide**

1. **Introduction**
   1. Should the transcript be provided for authorization?
   2. Questions about the project or interview?

- Start recording
  1. Permission to record the interview
  2. Informed consent
  3. Confirmation of eligibility criteria
  4. Reminder: not every question has to be answered and the interview can be stopped at any time, please answer honestly

1. **Possible introductory questions**
   1. What are your tasks in working with people who have had a stroke?
   2. What do you normally do when working with people who have had a stroke?
   3. Please describe your work with people who have had a stroke.
2. **Key questions**
   1. What role do guidelines play in your daily work?

Presentation of the core recommendations of the “Rehabilitation of Mobility after Stroke (ReMoS)” guideline. These are referred to below as the "ReMoS recommendations".

- 1. What influences your use of the ReMoS guideline?
  2. What needs to be changed so that you implement the ReMoS recommendations (more often)?

1. **Closing questions**
   1. What content have we not yet talked about that is important to you?
   2. Would you like to add anything in conclusion?
   3. Do you have any questions for me?
2. **Demographic information**
3. **Facultative Prompts**
   1. Knowledge
      1. What sources of information do you use to find out about therapy measures?
      2. What access do you use to the ReMoS guideline?
      3. What can you tell me about the recommendations of the ReMoS guideline?
   2. Skills
      1. What skills do you need to be able to implement the recommendations of the ReMoS guideline?
      2. How easy or difficult is it to implement the ReMoS recommendations?
      3. What skills do you have to implement the ReMoS recommendations?
   3. Decision making
      1. On what basis do you make decisions about the treatment process?
      2. What helps you to remember the ReMoS recommendations?
   4. Behaviour Regulation
      1. Do you have to do anything before you can implement the ReMoS recommendations?
      2. Is there anything that makes you do it?
   5. Social influence
      1. What influence do other people (other health professionals, doctors, patients, relatives, etc.) have on how you implement the ReMoS recommendations?
      2. How do your colleagues implement the ReMoS recommendations?
      3. Do you feel pressured by anyone when implementing the ReMoS recommendations?
   6. Environment and resources
      1. Do you have the necessary equipment at your workplace to implement the ReMoS recommendations?
      2. Who supports you in/stops you from implementing the ReMoS recommendations?
      3. Do processes in your work environment need to be changed in order to implement the ReMoS recommendations (more often)?
   7. Professional role
      1. What is your role in implementing the ReMoS recommendations?
      2. What is the role of other professionals in your work environment?
      3. Who should be involved in the implementation of the ReMoS recommendations?
   8. Belief in skills
      1. How confident do you feel in implementing the ReMoS recommendations?
      2. What do you do particularly well in terms of implementing the ReMoS recommendations?
      3. What do you find difficult about implementing the ReMoS recommendations?
   9. Optimism
      1. How useful are the ReMoS recommendations in your daily work?
      2. How does the implementation of the ReMoS recommendations influence the care of your patients/your daily work?
      3. Do you sometimes worry about missing something?
   10. Assumptions about consequences
       1. What would happen if you implemented the ReMoS recommendations (more often)?
       2. What are the positive/negative consequences for you of implementing the ReMoS recommendations?
       3. What are the advantages/disadvantages for your patients/your daily work if you implement the ReMoS recommendations?
   11. Intentions
       1. What are your intentions in implementing the ReMoS recommendations?
       2. How often would you like to implement the ReMoS recommendations?
   12. Emotions
       1. How do you feel about implementing the treadmill/gait trainer training with stroke patients?
       2. What is it like for you to do endurance/intensive gait training with the stroke patient?
       3. How do feelings influence whether you implement the ReMoS recommendations?
   13. Reinforcement
       1. What strategies would you recommend to promote the implementation of the ReMoS recommendations in your workplace?
       2. What could improve the use of the ReMoS guideline?
   14. Goals
       1. What should your work with patients who have had a stroke look like in the next 10 years?
       2. What role will the ReMoS guideline have in your daily work in the future?
